# Evaluation of various membranes for blood-feeding in nine sand fly species and artificial feeding challenges in *Sergentomyia minuta*

**DOI:** 10.1186/s13071-025-06729-8

**Published:** 2025-03-27

**Authors:** Anna Hošková, Barbora Vojtková, Markéta Stejskalová, Nikola Polanská, Magdalena Jančářová, Lidiane Medeiros da Costa, Mauricio Roberto Viana Sant´Anna, Petr Volf, Jovana Sádlová

**Affiliations:** 1 Charles University, Prague, Czech Republic; 2 Universidade Federal de Minas Gerais, Belo Horizonte, Brazil

**Keywords:** *Sergentomyia minuta*, *Phlebotomus*, *Lutzomyia*, Vector competence, Artificial feeding, *Leishmania*

## Abstract

**Background:**

We evaluated various membranes for blood-feeding in nine sand fly species from different genera and subgenera. Most of these species are vectors of human-pathogenic *Leishmania*, whereas *Sergentomyia minuta* is a herpetophilic sand fly species and a proven vector of *Leishmania* (*Sauroleishmania*) *tarentolae.*

**Methods:**

Female sand flies were offered blood through a range of membranes (chicken, reptilian, and frog skin; synthetic collagen; pig intestine; and duck foot webbing). Two feeding systems (glass feeder, Hemotek) and different blood sources (human, ovine, avian, and reptilian) were used. Feeding trials were conducted under varying thermal and light conditions to determine the optimal parameters.

**Results:**

Among the 4950 female *S. minuta* tested, only a negligible fraction took a blood meal: 2% of the females fed on avian blood, and 0.2% of the females fed on human blood. In eight other species, the chicken membrane was generally more effective than synthetic membranes or pig intestines. For example, *Phlebotomus duboscqi* refused synthetic membranes, while *Lutzomyia longipalpis* and *P. perniciosus* avoided both synthetic membranes and pig intestines. The most effective membrane was duck foot webbing, with four species feeding more readily through it than through the chicken membrane. Additionally, applying coagulated blood plasma to the outer surface of chicken or synthetic membranes significantly increased feeding rates.

**Conclusions:**

Female *S. minuta* did not reliably feed on blood through the tested membranes, preventing laboratory infection experiments from confirming their vector competence for human-pathogenic *Leishmania*. However, for future experimental infections of other sand fly species, duck foot webbing has emerged as an effective membrane, and the application of blood plasma to the exterior of membranes may increase the feeding rates.

**Graphical abstract:**

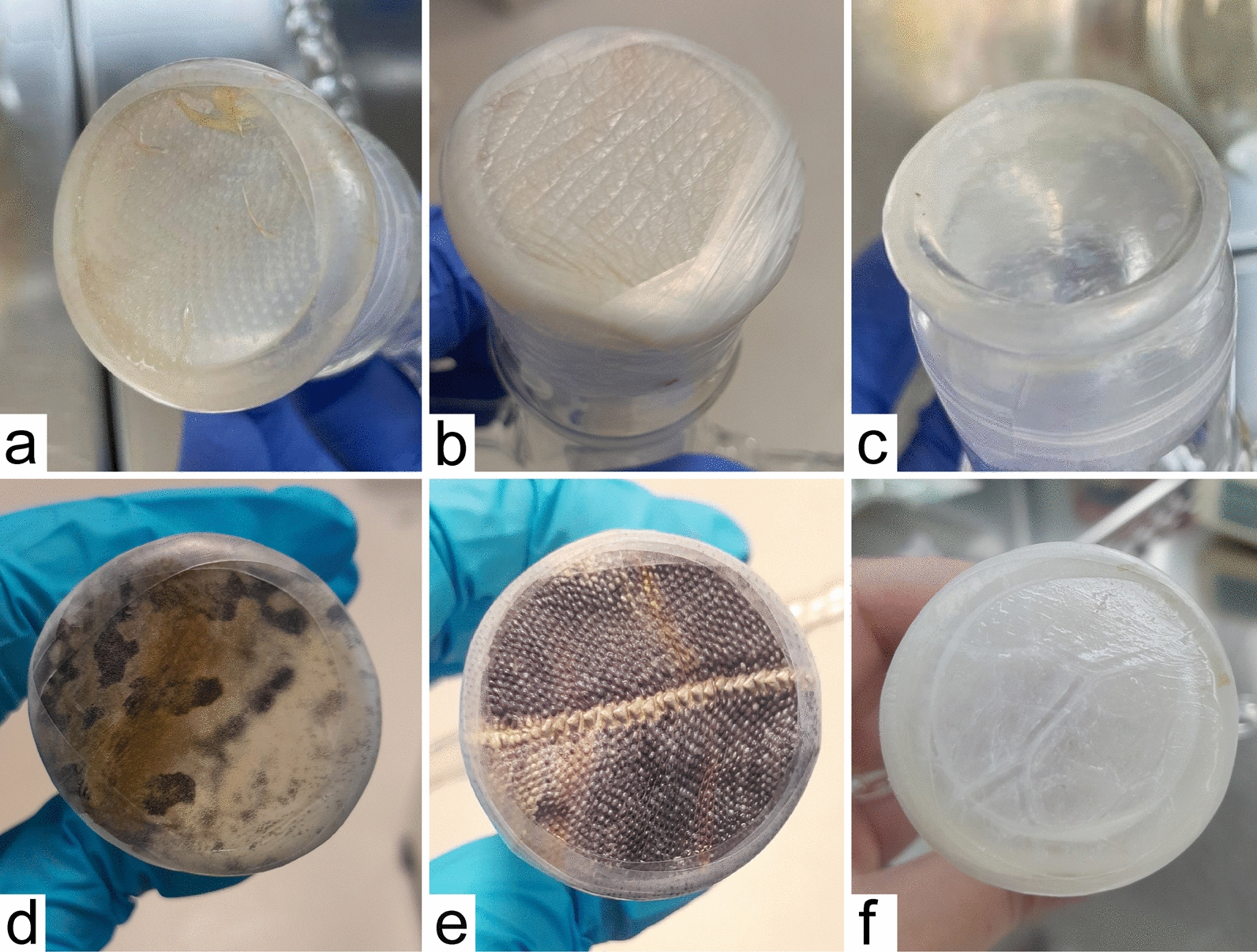

## Background

Phlebotomine sand flies (Diptera: Psychodidae, Phlebotominae) are tiny hematophagous insects found mainly in tropical and subtropical areas. Approximately 1000 species have been described globally, with approximately 100 species proven or suspected as vectors of *Leishmania* parasites, *Bartonella,* and sand fly-borne viruses [1, 2]. Both sexes of adult sand flies feed on plant nectar, but females also require blood meals to obtain essential nutrients for egg production.

In the Old World, only sand flies of the genus *Phlebotomus* are confirmed vectors of *Leishmania* parasites that are pathogenic to humans. Conversely, sand flies of the genus *Sergentomyia,* which are widely distributed throughout the Old World, are primarily herpetophilic and act as proven vectors of reptile *Leishmania* (*Sauroleishmania*) species [3]). In recent years, however, the extensive application of molecular biology methods to field-caught sand flies has led to the discovery of many mammalian *Leishmania* spp. in various representatives of this genus (reviewed in [4]). Among these, *Sergentomyia minuta*, one of the most abundant sand fly species in the Mediterranean, is a proven vector of *Leishmania* (*Sauroleishmania*) *tarentolae*, a non-pathogenic parasite of reptiles [5, 6]. Although *S. minuta* preferentially feeds on reptiles [7], numerous studies have documented occasional feeding on humans and other mammals [7–14]. In addition, *S. minuta* has often been collected from endemic foci of leishmaniasis, where it was found infected with *Leishmania major* [15], *Leishmania infantum* [13, 14, 16], or Toscana virus [17, 18].

These findings suggest that *S. minuta* could contribute to the transmission of *Leishmania* species that are pathogenic to humans (see [4] for a review). However, the detection of *Leishmania* DNA in a female sand fly does not necessarily confirm vector competence. The early phase of infection is non-specific, as *Leishmania* can temporarily survive in the digested blood meal of various sand fly species and other blood-feeding arthropods. However, only competent vectors retain the infection beyond defecation and successfully transmit it to vertebrate hosts [19, 20]. Therefore, experimental infections and host-transmission experiments are needed to verify the vector competence of *S. minuta.*

Generally, experimental infections of *Sergentomyia* species are challenging. While dozens of species of the genera *Phlebotomus* and *Lutzomyia* have been colonized and much experience with their membrane-feeding ability has accumulated [21], representatives of the genus *Sergentomyia* that preferentially feed on cold-blooded vertebrates have rarely been colonized [22, 23]. Their rearing usually requires blood-feeding on reptiles, and the most challenging step is inducing females to feed on infectious blood, as not all species are able to feed through the usual chicken skin membrane [24].

This study aimed to test several types of membranes, blood sources, feeding systems, and experimental conditions suitable for testing the vector competence of *S. minuta*. The selection of membranes includes the most commonly used materials for artificial feeding of phlebotomine sand flies or mosquitoes (chicken skin, synthetic collagen membrane, pig intestine), but owing to the feeding preferences of *S. minuta*, we have also included reptile skin and two new materials (frog skin, duck foot webbing). Eight other sand fly species with known vectorial competence were tested for comparison.

## Methods

### Sand flies

Laboratory colonies of *S. minuta* (originally from Portugal), *Sergentomyia schwetzi* (originally from Ethiopia), *Lutzomyia longipalpis* (originally from Brazil), *Lutzomyia migonei* (originally from Brazil), *Phlebotomus perniciosus* (originally from Spain), *Phlebotomus duboscqi* (originally from Senegal), *Phlebotomus arabicus* (originally from Israel), *Phlebotomus sergenti* (originally from Turkey), and *Phlebotomus argentipes* (originally from India) were maintained in the insectary of Charles University in Prague under standard conditions (26 °C, humidity in the insectary 60–70%, photoperiod 14 h light/10 h dark, fed with 50% sucrose) as described previously [25].

### Sand fly membrane feeding

The experiments were conducted in a 150 × 150 cm box under insectary conditions, with a stable temperature of 26 °C and humidity (50–60%) (unless otherwise specified), or in a glove box. Commercially available defibrinated ovine blood (LabMedia, s.r.o.), which is a common standard for infections of other sand fly species in our laboratory, was used for most experiments. Since *S. minuta* is a herpetophilic species that sometimes feeds on humans, we also used reptile blood (heparinized blood of *Eryx colubrinus*, obtained from the Department of Zoology, Charles University, as surplus material collected within the project performed with the permission of the Ministry of Education, Youth and Sports of the Czech Republic), related bird blood (citrated chicken blood, Biopharm), and heparinized human blood (JS).

**Fig. 1:**
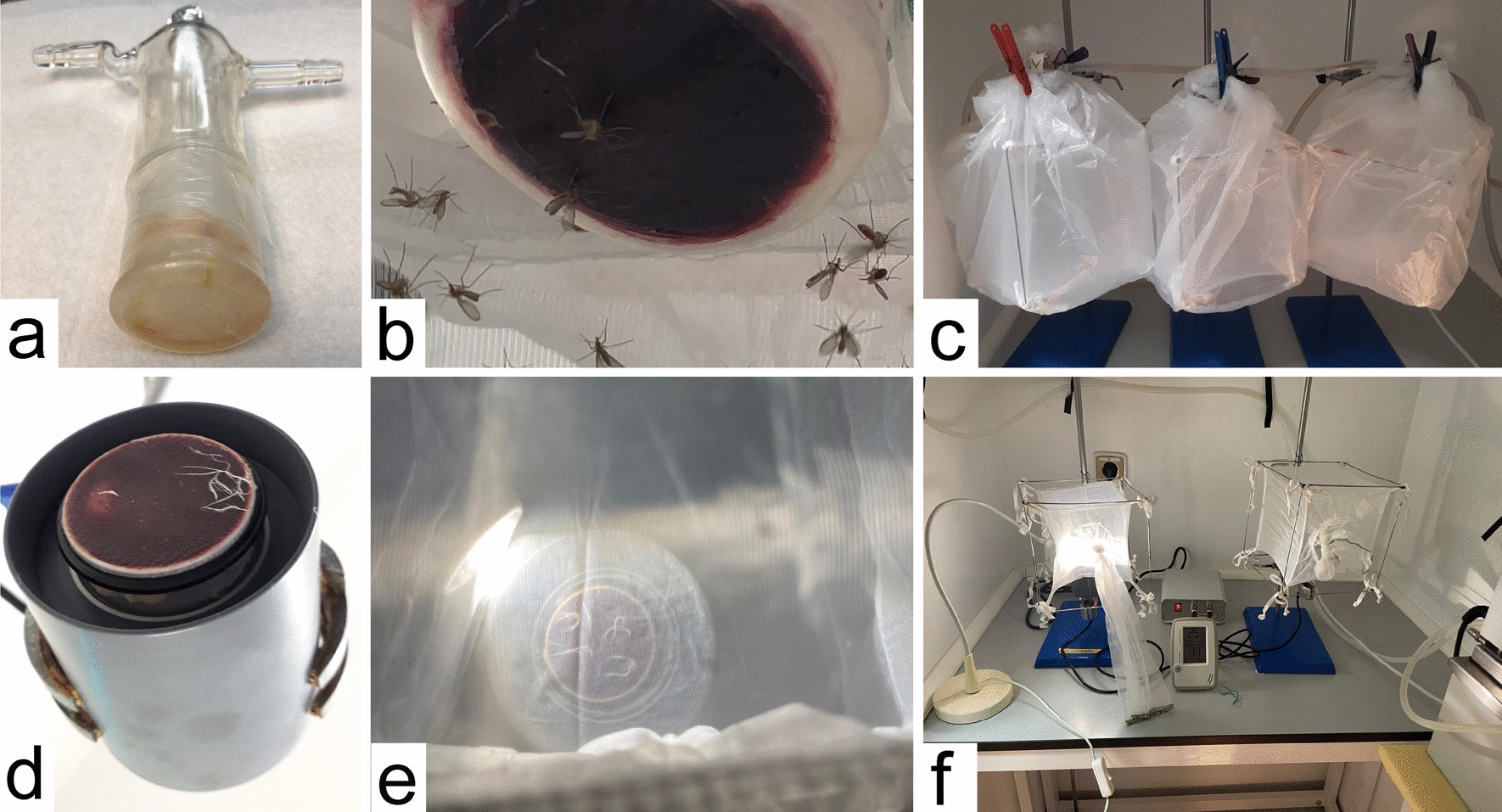
Artificial feeding systems for sand flies. **a** Glass feeder with attached chick skin membrane; **b** glass feeder filled with blood inside the cage with sand flies; **c** sand flies feeding on glass feeders connected to the water bath; **d** Hemotek with attached chick skin membrane; **e** Hemotek under the cage; **f** feeding of sand flies via the Hemotek system

The female sand flies were exposed to membrane-feeding via either a commercially available Hemotek system or a glass feeder, as described previously [25]. The preparation of membranes is described below. The membranes were fixed to the glass feeder using Parafilm, and the feeder was filled with blood and connected to circulating heated water. The nylon net with sand flies was attached with a rubber band to the underside of the feeder, and the plastic bag holding the moisture was fastened with pins (Fig. 1a–c). The Hemotek system was assembled according to the manufacturer’s instructions. The metal reservoir was covered with a chicken skin fixed with a rubber band and filled with blood. The reservoir was mounted on a Hemotek set at 37 °C, and a net with sand flies was placed on top of the reservoir (Fig. 1d–f). In both systems, the sand flies were allowed to feed for 1.5–2 h unless otherwise specified, and all the experiments were performed in duplicate.

In certain experiments, chicken skin or synthetic membranes were coated with dried coagulated blood plasma (CBP). Ram blood was centrifuged at 4380 rpm for 10 min, after which the supernatant was collected and heated in boiling water. Once the plasma had solidified into a gel-like structure, it was placed in a Petri dish, and a membrane fixed on a glass feeder was immersed in this gel. Before the setup was exposed to sand flies, the gel coating on the membrane was dried via a hair dryer. In additional experiments, gecko feces were placed near the feeder to serve as an olfactory stimulus. All the experimental variables are summarized in Table 1.

**Table 1:** Summary of experimental variables

Membrane types	Blood sources	Feeding systems	Additional stimuli	Other factors
Chicken skin	Ovine	Glass feeder	Gecko feces	Environment (insectary vs. glove box)
Pig intestine	Avian (chicken)	Hemotek	CBP	Blood temperature
Synthetic (Hemotek Ltd.)	Reptile (*Eryx colubrinus*)			Circadian phase
Duck foot webbing	Human			Humidity
Reptile skin				
Frog skin				

### Preparation of membranes

*Chicken skin.* The feathers were removed from the skin of 1–3-day-old chicks (Agro-Bio s.r.o.). The skin was separated from the dorsal and ventral sides of the chicken and washed successively in sterile saline for 5 min, 70% pure ethanol for 5 min, and sterile saline for 5 min, and stored at −20 °C.

*Pig intestine*. Salted pig intestines, which are commercially available for sausage production, were macerated at 4 °C in distilled water for 48 h and in sterile saline for 48 h, with washing every 12 h to remove excess salt. The intestines were subsequently cut into small fragments (4 × 4 cm), which were again stored at 4 °C in saline for 72 h and then washed every 12 h. Finally, the pieces were spread flat in Petri dishes and stored at −20 °C.

*Synthetic Hemotek feeding membrane (Hemotek Ltd)*. The membrane was cut into pieces (4 × 4 cm) and washed in the same manner as the chicken skin. The membrane was utilized for blood-feeding immediately, as it was prone to rupture after storage at −20 °C.

*Duck foot webbing*. Duck feet from fresh cadavers were obtained from the Laboratory of Helminthology, Department of Parasitology, Faculty of Science, Charles University, as surplus material from a project performed with the permission of the Ministry of Education, Youth and Sports of the Czech Republic. Webbing has two layers that must be separated. The individual layers were washed and stored at −20 °C.

*Reptile skin.* Fresh cadavers of chameleons and geckos were obtained from a private owner. The skin was removed from the dorsal and ventral sides of the body, washed, and stored at −20 °C.

*Frog skin*. A fresh *Pelophylax ridibundus* cadaver was obtained from the field parasitology course taught at the Department of Parasitology, Faculty of Science, Charles University, as surplus material of the project performed with the permission of the Ministry of Agriculture of the Czech Republic. The skin was dissected from the dorsal side of the frog, washed, and stored at −20 °C.

### Statistical analysis

Differences in feeding rates among sand fly groups subjected to various feeding methods were analyzed using Pearson's Chi-square test and *z*-test in SPSS software (version 27, IBM Corporation, Armonk, NY, USA).

## Results

### Feeding of *S. minuta* on different blood sources via a glass feeder and chicken skin

The results of feeding *S. minuta* on three different blood sources through the chicken skin membrane under varying conditions of circadian phase, blood temperature, humidity, light alteration, and olfactory stimuli are summarized in Table 2. Females were exposed to blood in 10 replicates (three for avian blood, four for reptile blood, and three for human blood). They fed successfully under only two conditions: three out of 150 females fed at night on avian blood maintained at 32 °C with high humidity in the glove box using gecko excrement as an olfactory stimulus, and one out of 450 females fed during the daytime on human blood maintained at 37 °C in the insectary, also with gecko excrement as an olfactory stimulus (Table 2).

**Table 2:** Feeding rates of *S. minuta* on different blood sources using glass feeder and chicken skin

Circadian phase	Experimental design	Blood source	Feeding rate (%)
Day	G, BT 26 °C	Avian (chicken)	0/150 (0%)
Day	G, BT 32 °C, high humidity, OS	Avian (chicken)	0/150 (0%)
Night	G, BT 32 °C, high humidity, OS	Avian (chicken)	3/150 (2%)
Day	G, BT 30–37 °C, OS, L/D	Reptile (*Eryx colubrinus*)	0/120 (0%)
Night	G, BT 30–37 °C, OS, L/D	Reptile (*Eryx colubrinus*)	0/120 (0%)
Day	I, BT 30–37 °C, OS, L/D	Reptile (*Eryx colubrinus*)	0/120 (0%)
Night	I, BT 30–37 °C, OS, L/D	Reptile (*Eryx colubrinus*)	0/120 (0%)
Day	I, BT 37 °C, OS, L/D	Human	1/450 (0.2%)

*G* glove box, *I* insectary, *BT* blood temperature, *OS* gecko excrement as an olfactory stimulus, *L/D* alternation of light and dark during feeding

### Feeding of *S. minuta* through different types of membranes via a glass feeder

**Fig. 2:**
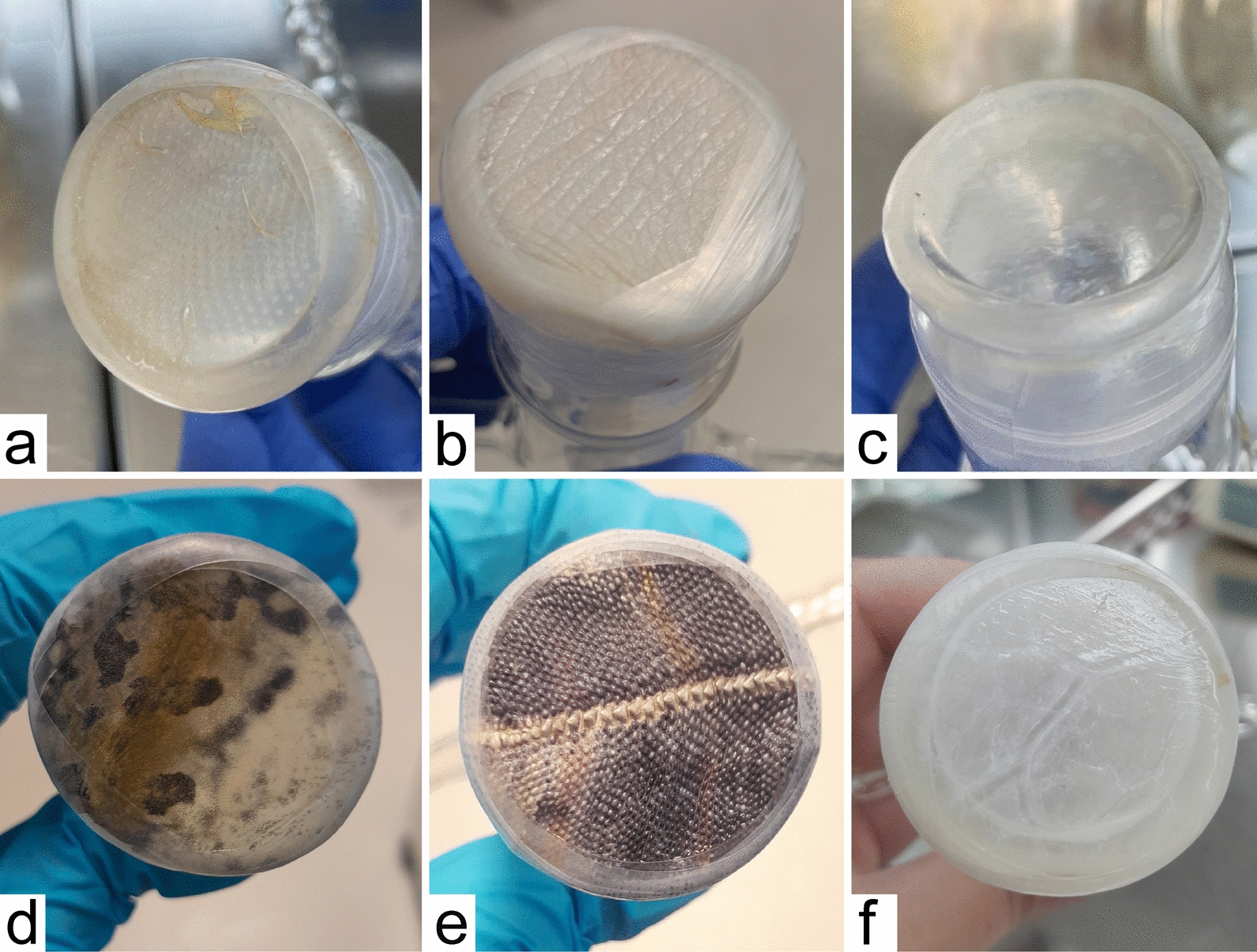
Types of membranes used for the feeding of *S. minuta*. **a** chicken skin; **b** duck foot webbing; **c** synthetic membrane; **d** frog skin; **e** reptile skin; **f** pig intestine

Owing to the reluctance of *S. minuta* to feed through chicken skin, we also tested other types of membranes — synthetic membrane, pig intestine, duck foot webbing, reptilian skin, and frog skin (the appearance of the individual membranes is presented in Fig. 2). Out of a total of 2360 *S. minuta* females exposed to blood-feeding in 21 replicates (seven for reptile skin and two for each of other membrane types), none successfully fed on blood (Table 3). These experiments were conducted in the daytime with a glass feeder filled with ram blood heated to 37 °C, in a darkened room in the insectary. In some experiments with chicken skin and synthetic membranes, dried CBP was added, but the feeding rate did not increase (Table 3). In the experiments with reptile skin, the blood temperature was varied between 25 °C and 37 °C, and the feeding time was between 1 and 3.5 h, but again, there was no significant effect on the feeding rates (Table 4).

**Table 3:** Feeding rates of *S. minuta* through different types of membranes with glass feeder and ram blood

Type of membrane	Feeding rate (%)
Chicken skin	0/200 (0%)
Chicken skin with CBP	0/200 (0%)
Synthetic membrane	0/200 (0%)
Synthetic membrane with CBP	0/150 (0%)
Pig intestine	0/200 (0%)
Duck foot webbing	0/180 (0%)
Reptile skin	0/1050 (0%)
Frog skin	0/180 (0%)

**Table 4:** Feeding rates of *S. minuta* through reptile skin using glass feeder and ram blood

Membrane type	Temperature of blood	Duration of feeding	Feeding rate (%)
Chameleon skin	37 °C	1 h	0/200 (0%)
Chameleon skin	25–37 °C	3.5 h	0/200 (0%)
Chameleon skin	25–37 °C	1.5 h during evening	0/200 (0%)
Gecko skin	37 °C	2 h	0/450 (0%)

### Feeding of *S. minuta* through chicken skin via a Hemotek system

For experiments with the Hemotek system, ram blood heated to 37 °C was used. Feeding lasted for 2 h, and none of the 160 females consumed blood. This experiment was performed twice.

### Feeding of eight sand fly species through different types of membranes via a glass feeder

**Fig. 3:**
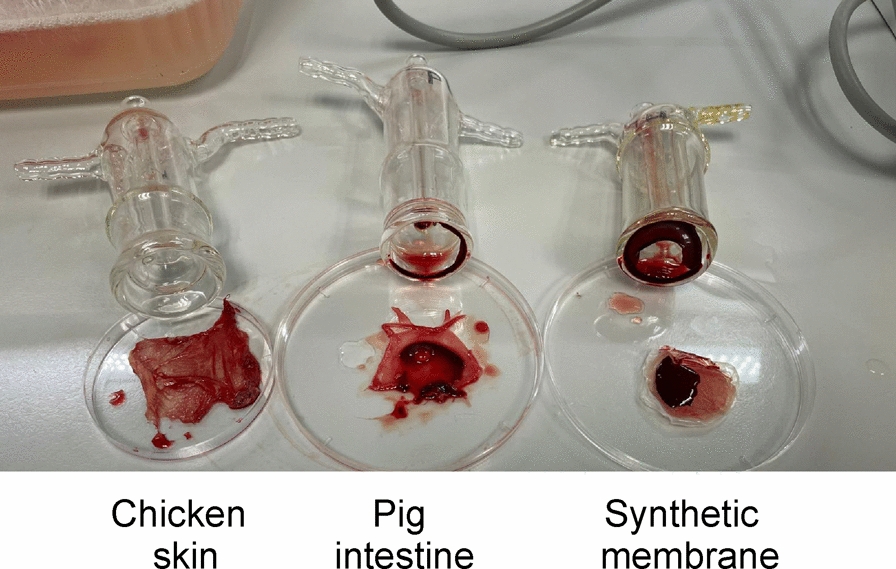
Appearance of blood after removing the membranes from glass feeders. The feeders were filled with the same ram blood and left for 1.5 h in a water bath heated to 37 °C

Feeding rates were also tested in various sand fly species representing different subgenera. Different membranes were compared, with chicken skin serving as the standard (Table 5). The females were exposed to blood in 74 replicates (two for each membrane type). Duck foot webbing was less successful than chicken skin for *L. migonei* and *P. perniciosus*, equally successful for *L. longipalpis* and *P. duboscqi*, and more effective for four species: *S. schwetzi*, *P. arabicus*, *P. sergenti*, and *P. argentipes*. For all the species, chicken skin significantly outperformed the synthetic membrane, which was particularly ineffective for *L. longipalpis*, *P. duboscqi*, and *P. pernicious,* where almost no feeding occurred. The pig intestine membrane had significantly lower feeding success than the chicken membrane in seven sand fly species, the exception being *P. argentipes*, where 72.2% of females fed on the pig membrane and 56.7% on the chicken membrane. The lower feeding rate observed with artificial membranes and pig intestines might be related to the fact that these membranes are more permeable, causing blood to thicken on the surface, as was evident when the membranes were removed from the feeders after the experiment (shown in Fig. 3).

**Table 5:** Feeding rates of sand flies through different types of membranes using a glass feeder

Sand fly species	Chicken skin	Chicken skin with CBP	Synthetic membrane	Synthetic membrane with CBP	Pig intestine	Duck foot webbing	Statistics (Chi-square test)
*S.* (*Sergentomyia*) *schwetzi*	61/150	174/200	23/140	Not done	40/140	81/150	*P* < 0.0001
	(40.7%)^a^	(87.0%)^b^	(16.4%)^c^		(28.6%)^d^	(54.0%)^e^	*df* = 4
							*χ*^2^ = 204.08
*L.* (*Lutzomyia*)* longipalpis*	183/200	Not done	1/200	0/200	2/200	159/180	*P* < 0.0001
	(91.5%)^a^		(0.5%)^b^	(0%)^b^	(1.0%)^b^	(88.3%)^a^	*df* = 4
							*χ*^2^ = 817.44
*L.* (*Migonemyia*) *migonei*	129/200	Not done	68/200	Not done	102/200	56/130	*P* < 0.0001
	(64.5%)^a^		(34.0%)^b^		(51.0%)^c^	(43.1%)^b,c^	*df* = 4
							*χ*^2^ = 39.35
*P.* (*Larroussius*) *perniciosus*	78/140	Not done	7/140	51/200	7/140	41/200	*P* < 0.0001
	(55.7%)^a^		(5.0%)^b^	(25.5%)^c^	(5.0%)^b^	(20.5%)^c^	*df* = 4
							*χ*^2^ = 139.50
*P.* (*Phlebotomus*) *duboscqi*	104/150	Not done	2/80	44/300	16/80	36/60	*P* < 0.0001
	(69.3%)^a^		(2.5%)^b^	(14.7%)^c^	(20.0%)^c^	(60.0%)^a^	*df* = 4
							*χ*^2^ = 201.85
*P.* (*Adlerius*)* arabicus*	118/190	Not done	30/190	Not done	22/190	106/130	*P* < 0.0001
	(62.1%)^a^		(15.8%)^b^		(11.6%)^b^	(81.5%)^c^	*df* = 4
							*χ*^2^ = 243.59
*P.* (*Paraphlebotomus*) *sergenti*	47/180	78/150	28/180	Not done	19/180	108/200	*P* < 0.0001
	(26.1%)^a^	(52.0%)^b^	(15.6%)^c^		(10.6%)^c^	(54.0%)^b^	*df* = 4
							*χ*^2^ = 136.45
*P.* (*Euphlebotomus*) *argentipes*	102/180	Not done	74/180	Not done	130/180	180/200	*P* < 0.0001
	(56.7%)^a^		(41.1%)^b^		(72.2%)^c^	(90.0%)^d^	*df* = 4
							*χ*^2^ = 110.58

*CBP* coagulated and dried blood plasma. The numbers indicate the number of fed females/total number of exposed females (feeding rate). Superscript letters indicate statistical differences between groups within the same sand fly species. Letters differ when differences in infection rates between feeding methods are statistically significant

*Lutzomyia migonei* and *P. argentipes* showed the most generalist feeding behavior, with more than 34% and 41% of females, respectively, feeding on all four membrane types. In contrast, *L. longipalpis* presented the most selective behavior, with 91% and 88% of the females feeding on chicken and duck membranes, respectively, whereas only 0.5% and 1.0% of the females fed on artificial membranes and pig intestines, respectively.

In species where less than 40% of the females were fed through the chicken membrane or where 5% or less were fed through the artificial membrane, CBP was applied to the membrane surface to enhance feeding. This significantly increased the feeding rates: from 41% to 87% in *S. schwetzi* and from 26% to 52% in *P. sergenti* fed through the chicken membrane. Similarly, feeding on artificial membranes increased from 5% to 25% in *P. perniciosus* females and from 2.5% to 15% in *P. duboscqi* females.

## Discussion

We compared various membranes for feeding eight *Phlebotomus* and *Lutzomyia* species, which are vectors of medically important *Leishmania* parasites. In addition, we aimed to develop an experimental method for feeding and infecting *S. minuta* sand flies to evaluate their vector competence for *Leishmania* parasites. A total of 4950 female *S. minuta* were fed through various membranes, on different blood sources, and under various experimental conditions.

*Sergentomyia minuta* females are considered primarily herpetophilic [26], but molecular analysis of blood meals from field-collected samples have revealed that they also feed on humans [9–14] and other mammals, such as *Mus musculus* [8], *Oryctolagus cuniculus* [11, 12], *Lepus granatensis* [11], *Bos taurus* [11, 12], *Sus scrofa* [12], *Canis lupus* [12], *Equus caballus* [12], and *Equus africanus* [12]. However, in our laboratory, a colony of *S. minuta* fed readily on lizards (*Podarcis siculus*) and geckos (*Tarentola mauritanica*, *Hemidactylus turcicus*) but rejected mice and rabbits, with only 3% feeding on humans [7]. As direct feeding on infected mammals was not feasible, we attempted to introduce infections via membrane feeding. We offered them different blood sources in a glass feeder covered with chicken skin, but *S. minuta* completely refused to feed on ram and reptile blood; only three females out of 450 fed on avian blood, and only one female out of 450 fed on human blood. Manipulating blood temperature, changing the environment (glove box vs. insectary), humidity, lighting, or time of day, or adding gecko excrement as an olfactory stimulus did not help.

We also tested the Hemotek system, where the reservoir filled with blood can be situated at the bottom of the net, allowing sand flies to feed in a more natural position (while on the glass feeder, the females have to feed upside down). Studies on biting midges have shown that this design positively affects the feeding rate of *Culicoides imicola* females [27], but it was not successful in our experiments with *S. minuta*.

The next option was to modify the membrane covering the feeder. Chicken skin is the most commonly used membrane for experimental blood-feeding of phlebotomine sand flies, and many studies indicate that it is more effective than other membranes [25, 28–31]. The use of chicken skin with a glass feeder and ram blood is also a standard method in our laboratory. However, for *S. minuta*, we were forced to explore other options and compare the effectiveness of different types of membranes with eight other sand fly species.

Synthetic membranes have been used in the research and maintenance of mosquitoes and other blood-feeding insect colonies (reviewed in [32, 33]). In our study, all the sand fly species tested except *S. minuta* consumed blood through a synthetic collagen membrane, but the feeding rates were significantly lower than those through standard chicken skin. Specifically, *L. longipalpis*, *P. perniciosus*, and *P. duboscqi* had negligible feeding rates (less than 5%) on the synthetic membrane. This mirrors findings in mosquitoes, where the efficacy of animal-derived membranes has been shown to be greater than that of the synthetic membranes [34–37].

Among other animal materials, pig intestines have been successfully used for feeding sand flies [38, 39]. In our experiments, only female *P. argentipes* fed better through the pig intestine than through the chicken skin, whereas seven sand fly species fed at a significantly lower percentage than through the chicken membrane. Specifically, *L. longipalpis* and *P. perniciosus* fed at less than 5%, and female *S. minuta* again refused to feed completely. In both the intestine and synthetic membranes, we observed clotted blood on the inner side of the membrane and inside the glass feeder (Fig. 3). This clotting may hinder feeding and explain the low feeding rates of some of the tested groups of sand flies.

To our knowledge, duck foot webbing was tested here for the first time. It was proven to be an excellent alternative to chicken skin. All the sand fly species tested (except *S. minuta*) consumed the blood meal, and the feeding rates were comparable or even significantly higher than those observed with standard chicken skin. Since female *S. minuta* preferentially feed on reptiles [7], we also tested skins from chameleons and geckos. More than 1000 females of *S. minuta* were allowed to feed through these reptile skins, but none of them consumed blood. Additionally, experiments with frog skin were unsuccessful. To increase the attractiveness of the membranes, CBP was applied to the outer surface of both the chicken skin and the synthetic membrane, offering them to *S. minuta* and other species with low feeding rates. This significantly increased the feeding rate, with the only exceptions being *L. longipalpis* on the synthetic membrane and, again, *S. minuta*.

Although data on the laboratory behavior of sand flies of the genus *Sergentomyia* are generally scarce, the reluctance of *S. minuta* to feed on membranes is exceptional, even for this herpetophilic genus. In a search for *L. donovani* vectors among Kenyan sand flies, Kaddu et al. [24] reported that five *Sergentomyia* species could feed through chicken skin membranes: more than 90% of *S. adleri* females*,* more than 30% of *S. ingrami* and *S. schwetzi* females, and more than 5% of *S. antennatus* and *S. garnhami* females. Only *S. bedfordi* required a lizard skin membrane. Notably, *S. schwetzi* has been repeatedly used in vector competence experiments via membrane feeding [40–42].

Under current conditions, testing the vector competence of *S. minuta* via membrane feeding is not feasible. However, ongoing development of membrane-feeding materials may provide solutions. [33, 43]. Additionally, time may offer hope: newly established colonies may become more adaptable. For example, freshly colonized *Phlebotomus tobbi* females initially refused to feed on mice or artificial membranes, unlike most other sand fly species maintained in the Prague insectary. After 7 years, however, sufficient numbers have adapted, allowing vector competence studies [44].

## Conclusions

The reluctance of *S. minuta* to feed on artificial feeders currently prevents the testing of the vector competence of *S. minuta* to human pathogens through experimental infections. Long-term efforts to adapt *S. minuta* colonies to artificial feeding are needed. Alternatively, hybrid feeding approaches (e.g., combining natural hosts and artificial systems) should be used, but unfortunately, this approach is so far only applicable for research on reptilian *Leishmania* species. For the other phlebotomine sand fly species, this study demonstrated that all exposed species fed readily through duck foot webbing, with four species achieving even higher feeding rates than with the standard chicken membrane. Duck foot webbing is therefore a valuable alternative for the membrane feeding of sand flies and potentially other blood-feeding insects. On the other hand, synthetic membranes and pig intestines are less attractive to sand flies, and certain species refuse to feed through these materials. The feeding rates can be increased by applying CBP to the exterior of the membrane.

## Data Availability

No datasets were generated or analyzed during the current study.
